# Body mass and growth rates in captive chimpanzees (*Pan troglodytes*) cared for in African wildlife sanctuaries, zoological institutions, and research facilities

**DOI:** 10.1002/zoo.21718

**Published:** 2022-07-11

**Authors:** Bryony A. Curry, Aimee L. Drane, Rebeca Atencia, Yedra Feltrer, Glyn Howatson, Thalita Calvi, Christopher Palmer, Sophie Moittie, Steve Unwin, Joshua C. Tremblay, Meg M. Sleeper, Michael L. Lammey, Steve Cooper, Mike Stembridge, Rob Shave

**Affiliations:** ^1^ International Primate Heart Project, Cardiff School of Sport and Health Sciences Cardiff Metropolitan University Cardiff UK; ^2^ Centre for Heart, Lung, and Vascular Health, School of Health and Exercise Sciences University of British Columbia Okanagan Kelowna British Columbia Canada; ^3^ Cardiff School of Sport and Health Sciences Cardiff Metropolitan University Cardiff UK; ^4^ Tchimpounga Chimpanzee Sanctuary Jane Goodall Institute Pointe Noire Republic of Congo; ^5^ Faculty of Health and Life Sciences Northumbria University Newcastle‐upon‐Tyne UK; ^6^ Water Research Group, School of Environmental Sciences and Development Northwest University Potchefstroom South Africa; ^7^ Chimfunshi Wildlife Orphanage Chingola Zambia; ^8^ Biological Science University of New South Wales Sydney New South Wales Australia; ^9^ Tacugama Chimpanzee Sanctuary Freetown Sierra Leone; ^10^ College of Life and Environmental Sciences University of Birmingham Birmingham UK; ^11^ Department of Small Animal Clinical Sciences University of Florida College of Veterinary Medicine Gainesville FL USA; ^12^ Alamogordo Primate Facility Holloman AFB Alamogordo New Mexico USA

**Keywords:** growth, maturation, sexual dimorphism

## Abstract

Captive chimpanzees (*Pan troglodytes*) mature earlier in body mass and have a greater growth rate compared to wild individuals. However, relatively little is known about how growth parameters compare between chimpanzees living in different captive environments. To investigate, body mass was measured in 298 African sanctuary chimpanzees and was acquired from 1030 zoological and 442 research chimpanzees, using data repositories. An analysis of covariance, adjusting for age, was performed to assess same‐sex body mass differences between adult sanctuary, zoological, and research populations. Piecewise linear regression was performed to estimate sex‐specific growth rates and the age at maturation, which were compared between sexes and across populations using extra‐sum‐of‐squares *F* tests. Adult body mass was greater in the zoological and resarch populations compared to the sanctuary chimpanzees, in both sexes. Male and female sanctuary chimpanzees were estimated to have a slower rate of growth compared with their zoological and research counterparts. Additionally, male sanctuary chimpanzees were estimated to have an older age at maturation for body mass compared with zoological and research males, whereas the age at maturation was similar across female populations. For both the zoological and research populations, the estimated growth rate was greater in males compared to females. Together, these data contribute to current understanding of growth and maturation in this species and suggest marked differences between the growth patterns of chimpanzees living in different captive environments.

## Highlights

- African sanctuary chimpanzees are lighter compared with their zoological and research counterparts. Additionally, sanctuary chimpanzees have a slower growth rate compared to zoological and research populations, and male sanctuary chimpanzees have an older age at maturation.
- Male sanctuary chimpanzees have a slower growth rate and an older age at maturation compared to zoological and research males.

## Graphical abstract

Piecewise linear regression estimated that male chimpanzees (*Pan troglodytes*) housed in African sanctuaries had a slower rate of growth and an older age at maturation for body mass compared with their zoological and research counterparts. In contrast, male zoological and research populations had a similar growth rate and age at maturation.

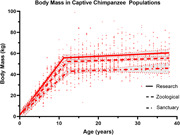

## 1 INTRODUCTION

Obesity is related to a multitude of comorbidities in captive chimpanzees (*Pan troglodytes*), including hypertension (Andrade et al., 2011; Ely et al., 2013; Videan et al., 2007), insulin resistance (Andrade et al., 2011), cardiovascular disease (Seiler et al., 2009; Strong et al., 2020), metabolic syndrome (Nunamaker et al., 2012; Steinetz et al., 1996) and inflammatory disease (Nehete et al., 2014; Obanda et al., 2014). Accordingly, appropriate management of body mass is an important consideration for the physical health and longevity of this species in captivity (Obanda et al., 2014). Successful management of body mass in captive chimpanzees, however, requires a comprehensive understanding of the normative growth pattern. Such data have only been well‐characterized in research populations, which have shown that females are typically lighter and attain body mass maturation earlier than males (Gavan, 1953; Grether & Yerkes, 1940; Hamada et al., 1996; Leigh & Shea, 1996). In contrast, comparatively few reports have examined the body mass of zoological (Vančata & Vančatová, 2002) or sanctuary (Cole et al., 2020; Obanda et al., 2014) populations. Nonetheless, a recent comparison between research chimpanzees and those living in African sanctuaries has identified the latter have a lower body mass and a slower rate of weight gain before maturation of body mass (Cole et al., 2020). However, it is currently unknown how the growth characteristics of zoological chimpanzees compare to that of research or sanctuary populations.

Growth is influenced by numerous factors, including physical activity and diet (Rogol et al., 2000) which vary across captive living environments (i.e., zoological institutions, research facilities, and African sanctuaries). In many of the sanctuaries in Africa, chimpanzees have access to large forested enclosures 10–100 times the size of the largest zoological (Wobber & Hare, 2011) or research enclosure. The smaller enclosure size in both zoological and research facilities may translate into lower physical activity levels, which in turn, could result in an earlier onset of maturation as has previously been documented in humans (Bacil et al., 2015). Further, a staple portion of the zoological and research chimpanzee diet is commercial monkey biscuit, which is of higher caloric density than native vegetation (AZA Ape TAG, 2010) that sanctuary chimpanzees primarily consume. The size and composition (i.e., male to female ratio and hierarchy) of social groupings also vary across the different captive environments, and accordingly, within‐group competition for food is likely to vary (Markham & Gesquiere, 2017). Groups in African sanctuaries can contain up to 50 individuals, and as greater group sizes are associated with complex social hierarchies that have increased competition for resources (Markham & Gesquiere, 2017), competition for food is likely to be greater in African sanctuaries compared with zoological and research facilities, where group size is smaller (e.g., Andrade et al., 2011; Birkett & Newton‐Fisher, 2011; Nunamaker et al., 2012; Videan et al., 2007). Consequently, the variations in diet, social grouping, and physical activity across captive living environments could influence adult body mass, the growth rate, and/or the timing of body mass maturation (i.e., asymptotic adult body mass). The aims of this study were, therefore, two‐fold (i) to compare adult body mass, growth rates, and ages at maturation for body mass between sanctuary, zoological, and research chimpanzees; (ii) to compare these growth parameters between sexes, within each population. It was hypothesized that in comparison to their zoological and research counterparts, sanctuary chimpanzees would be lighter, have a slower rate of growth, and have an older estimated age at body mass maturation. Additionally, it was hypothesized that across all three populations, body mass would be greater, and maturation would be attained at an older age, in males compared with their female counterparts.

## 2 MATERIALS AND METHODS

### 2.1 Sanctuary population

Single measurements of body mass were obtained in 298 chimpanzees (*P. troglodytes*) during routine health checks at three African rehabilitation sanctuaries (Tchimpounga Chimpanzee Rehabilitation Centre, Congo; Chimfunshi Wildlife Orphanage, Zambia; Tacugama Chimpanzee Sanctuary, Sierra Leone; Table 1). The three sanctuaries are members of the Pan African Sanctuary Alliance (PASA) and the chimpanzees were cared for in accordance with the recommendations of the PASA operations manual (Farmer et al., 2009). The majority of the chimpanzees (*n* = 252) were wild‐born orphans confiscated by wildlife authorities, commonly at the age of approximately 1–3 years, although some were older at arrival. The age of these individuals was estimated on arrival by highly experienced sanctuary veterinarians using dental development and records obtained during the confiscation (Cole et al., 2020; Wobber et al., 2010). For those chimpanzees born in captivity (*n* = 46), their precise age was used. Chimpanzees were housed in semi‐free ranging enclosures spanning from 2.5 to 77.0 hectares, in mixed‐sex and mixed‐age groups of 10–50 individuals. In addition to the native vegetation within the enclosures, the chimpanzees were supplemented routinely throughout the day with seasonal, locally obtained fruits and vegetables. While the subspecies were not known for every chimpanzee, the sanctuary population was likely to be of mixed subspecies; many chimpanzees at Tchimpounga were *P. t. troglodytes*, whereas the majority at Chimfunshi were thought to be *P. t. schweinfurthii* and those at Tacugama to be *P. t. verus*. Body mass was measured using either a calibrated hanging scale (Salter Brecknell, 235‐6S) or Seca electronic weighing scales (Seca, Vogel, and Halke) and was assessed to the nearest 0.1 kg. All procedures and protocols involved in this study have been endorsed by the PASA Advisory Council and Cardiff Metropolitan University, UK, approved by the British and Irish Association of Zoos and Aquariums and ethically approved by the University of British Columbia, Canada.

**Table 1:** Characteristics of the African sanctuary, zoological, and research populations of chimpanzee (*Pan troglodytes*)

Characteristic	Sanctuary				Zoological	Research
	CF	TAC	TCH	Combined		
Total (*n*)	107	60	131	298	1030	442
Male	50	25	76	151	409	196
Female	57	35	55	147	621	246
Age (years)						
Male	15 ± 9	14 ± 7	12 ± 7	14 ± 8	17 ± 9	12 ± 9
	(1–32)	(4–32)	(2–29)	(1–32)	(1–32)	(0–32)
Female	15 ± 8	16 ± 7	12 ± 5	15 ± 7	20 ± 11	15 ± 11
	(1–38)	(3–22)	(4–29)	(1–38)	(1–38)	(0–38)
Year(s) of data collection	2013 2018	2016	2015 2017	2013–2018	2000–2021	1980–2008

*Note*: Age (years) is presented as mean ± standard deviation (range)^a^.

^a^ CF: Chimfunshi Wildlife Orphanage, Zambia; TAC: Tacugama Chimpanzee Sanctuary, Sierra Leone; TCH: Tchimpounga Chimpanzee Rehabilitation Centre, Congo.

### 2.2 Zoological population

Anonymized body mass measurements from zoological chimpanzees were acquired from the Species360 Zoological Information Management System (2021), a comprehensive database that curates information recorded by a global network of zoological institutions. Measurements included in this analysis were obtained during health assessments completed between 2000 and 2021 in accredited zoological institutions across Europe and North America. Accredited institutions included those who were members of the World Association of Zoos and Aquariums (WAZA), or which held a WAZA‐affiliated association. These data were initially screened for obvious data input errors, and were then checked for outliers using the robust regression and outlier removal (ROUT) method (*Q* set to 1%) in GraphPad Prism (GraphPad Prism for Windows, version 8.0.1); however, this process did not identify any statistical outliers. To correspond with the age range of the African sanctuary population (0–32 years in males and 0–38 years in females), male and female zoological chimpanzees older than 32 years and 38 years of age, respectively, were excluded from the study. This was to ensure that the data sets were age comparable, and therefore any statistical findings were not due to differences in the age range between the populations. A single body mass measurement was randomly selected from each chimpanzee in the database, using the RAND function in Microsoft Excel (2016), to prevent any confounding effects of repeated measures. A total of 409 males and 621 females were included in the final analysis (Table 1). Unfortunately, no detailed information was available regarding the housing or diet of this population. Subspecies information was also not available for many of the individuals, however, the information that was available would suggest that, similar to the sanctuary population, the subspecies was mixed.

### 2.3 Research population

Publicly available body mass measurements from research chimpanzees were extracted from the Primate Aging Database (accessed November 2020; Primate Aging Database, 2019). This repository contains data from healthy, nonexperimental chimpanzees (Dansereau et al., 2019) housed at the Alamogordo Primate Facility, University of Texas M.D. Anderson Cancer Center and Yerkes National Primate Research Center, all of which were accredited by the Association for Assessment and Accreditation of Laboratory Animal Care International (AAALAC). Male chimpanzees over the age of 32 years, and female chimpanzees over the age of 38 years were excluded from the analysis to maintain comparable age ranges between populations. A single body mass measurement was randomly selected from each chimpanzee, using the same RAND function as described above. The total data set comprised of 196 males and 246 females (Table 1). All chimpanzees were socially housed, in either indoor (*n* = 226), outdoor (*n* = 116) or indoor with outdoor access (*n =* 100) enclosures. All chimpanzees received a diet of primate chow, supplemented with fruit and vegetables. Unfortunately, no information was available regarding the subspecies of this population.

### 2.4 Statistical analysis

Differences in mean adult body mass were assessed within sex across the three populations (i.e., sanctuary, zoological, and research) and between sexes within each population, using a two‐way analysis of covariance (ANCOVA) with Dunn–Sidak post hoc analyses, for which group and sex were independent variables and age was the covariate. The size of the effect was estimated using Cohen's *d*; here, *d* = ((*M*_1_–*M*_2_)/*s*_P_), where *M*_1_ = mean of group 1, *M*_2_ = mean of group 2, and *s*_P_ = pooled standard deviation (SD) between groups 1 and 2 (Cohen, 1988). An effect size of ≤0.2 was deemed a small effect, ≤ 0.5 a medium effect, and ≥0.8 a large effect. ANCOVA was performed using the Statistical Package for the Social Sciences version 26 (SPSS Inc.). Alpha was set at *p* < .05, and data were expressed as the mean difference (±SD) and 95% confidence intervals (CIs).

Growth rates and ages at maturation for body mass were estimated using sex‐specific piecewise least‐squares linear regressions in GraphPad Prism. An unconstrained analysis was chosen to model body mass and identify a pair of best fit lines and the breakpoint between these two lines (Altmann & Alberts, 2005). The slope of the regression line to the left of the breakpoint can be used as an estimate of growth rate (Altmann & Alberts, 2005; Huck et al., 2011) and the breakpoint as the estimated age at which maturation of body mass occurs (Leigh, 1994; Leigh & Terranova, 1998). This breakpoint was used to define the adult populations for the ANCOVA described above (i.e., those to the right of the breakpoint were considered adults). The extra‐sum‐of‐squares *F*‐test was used to determine whether growth rate and age at maturation differed across populations, or between sexes within a population.

## 3 RESULTS

### 3.1 Population differences in growth parameters

#### 3.1.1 Adult body mass

Mean adult body mass differed between the sanctuary, zoological and research populations (*p* < .001), following adjustment for age. In adult males, both the zoological (mean difference ± SD = 9.2 ± 10.1 kg, CI = 6.2–12.2 kg, *p* < .001, *d* = 0.92) and research (mean difference ± SD = 9.2 ± 9.9 kg, CI = 5.0–13.7 kg, *p* < .001, *d* = 1.26) populations had a greater body mass than the sanctuary chimpanzees (Table 2). However, adult body mass was similar between the male zoological and research populations (mean difference ± SD = 0.1 ± 10.0 kg, CI = −3.6–3.9 kg, *p* = .999; Table 2). In adult females, similar to males, both the zoological (mean difference ± SD = 10.0 ± 10.1 kg, CI = 7.2–12.8 kg, *p* < .001, *d* = 0.99) and research (mean difference ± SD = 18.1 ± 9.9 kg, CI = 13.3–22.8 kg, *p* < .001, *d* = 1.80) populations had greater body masses than sanctuary chimpanzees (Table 2). Additionally, female research chimpanzees had a greater adult body mass than their zoological counterparts (mean difference ± SD = 8.1 ± 9.9 kg, CI = 3.9–12.2 kg, *p* < .001, *d* = 0.80; Table 2).

**Table 2:** Body masses of the African sanctuary, zoological, and research populations of chimpanzee (*Pan troglodytes*) are reported for individuals of all ages, and for adults (defined as all measurements to the right of the estimated breakpoint, derived from the piecewise least‐squares linear regression)

Group	Sanctuary		Zoological		Research	
	*n*	Body mass (kg)	*n*	Body mass (kg)	*n*	Body mass (kg)
Males						
All	151	41.3 ± 16.4	409	50.0 ± 21.0	196	43.1 ± 24.7
		(4.0–74.9)		(2.7–97.0)		(1.6–91.8)
Adults	82	52.6 ± 8.1	266	61.8 ± 10.2	86	63.8 ± 10.1
		(32.0–74.9)		(33.5–97.0)		(50.0–86.0)
Adults (adjusted)^a^	82	53.0 ± 10.1^b^	266	62.3 ± 9.9^b^, ^c^	86	62.4 ± 10.0^c^
Females						
All	147	37.3 ± 11.9	621	47.2 ± 17.2	246	45.6 ± 20.9
		(4.3–64.7)		(3.0–96.0)		(1.5–91.5)
Adults	93	43.5 ± 7.5	444	54.7 ± 10.4	139	58.7 ± 12.7
		(25.2–64.7)		(34.9–96.0)		(38.0–96.5)
Adults (Adjusted)^b^	93	44.4 ± 10.1	444	54.4 ± 10.0^c^	139	62.4 ± 10.1^c^, ^d^

*Note*: Data presented are mean ± standard deviation (range).

^a^ Data reported are adjusted means for adults, controlling for age (years) as a covariate.

^b^ Significant sex difference within a population (*p* < .05).

^c^ Significant within sex difference versus sanctuary population (*p* < .05).

^d^ Significant difference versus zoological population (*p* < .05).

#### 3.1.2 Growth rates and ages at maturation

**Figure 1:**
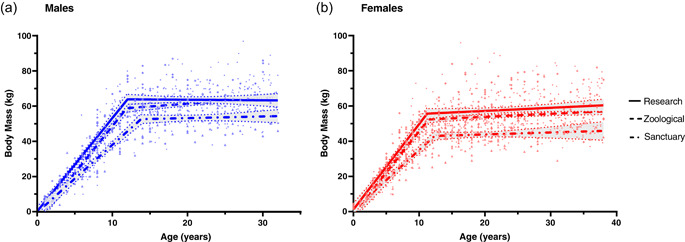
Comparison of body mass between male (a) and female (b) African sanctuary, zoological, and research populations of chimpanzee (*Pan troglodytes*). Body mass of sanctuary (represented by dot‐dashed lines), zoological (represented by dashed lines), and research chimpanzees (represented by solid lines) were fitted using piecewise least‐squares linear regression, with 95% confidence intervals shown (represented by gray area). Individual data points in the sanctuary, zoological, and research populations are represented by triangles, circles, and diamonds, respectively.  [Color figure can be viewed at wileyonlinelibrary.com]

Male sanctuary chimpanzees had a slower rate of body mass growth and attained body mass maturation at an older age compared with their zoological (*p* < .001 and *p* = .031, respectively) and research counterparts (*p* < .001 and *p* = .014, respectively; Figure 1 and Table 3). In contrast, male zoological and research populations had a similar growth rate and age at maturation (Figure 1 and Table 3). In females, sanctuary chimpanzees also had a slower rate of growth compared with their zoological (*p* = .018) and research counterparts (*p* = .007; Figure 1 and Table 3). The rate of growth was similar, however, between female zoological and research chimpanzees (Figure 1 and Table 3). Additionally, the age at maturation did not differ between the three female populations (Figure 1 and Table 3).

**Table 3:** Growth rate (kg yr^−1^; slope 1) and maturation age (years; breakpoint) of the African sanctuary, zoological, and research chimpanzee (*Pan troglodytes*) populations, as estimated using piecewise least‐squares linear regression

Regression parameter	Sanctuary zoological research					
	Male (*n *= 151)	Female (*n* = 147)	Male (*n* = 409)	Female (*n* = 621)	Male (*n* = 194)	Female (*n* = 243)
Slope 1	3.8	3.4	5.4^b^	4.7^a^, ^b^	5.3^b^	4.8^a^, ^b^
	(3.4–4.3)	(2.8–4.1)	(5.0–6.0)	(4.2–5.2)	(5.0–5.7)	(4.2–5.9)
Breakpoint	13.8	12.4	11.9^b^	11.4	12.0^b^	11.2
	(12.5–14.9)	(10.9–13.7)	(10.9–12.5)	(10.6–12.3)	(11.3–12.5)	(9.5–12.5)
*R* ^2^_adj_	0.81	0.69	0.80	0.70	0.9	0.79

*Note*: Data presented are the best fit value (95% confidence intervals).

^a^ Significant within sex difference versus sanctuary population (*p* < .05).

^b^ Significant sex difference within a population (*p* < .05).

#### 3.1.3 Sex differences in growth parameters

In both the sanctuary and zoological populations, male chimpanzees had a greater adult body mass compared with females (sanctuary: mean difference ± SD = 8.7 ± 10.1 kg, CI = 5.8–11.6 kg, *p* < .001, *d* = 0.86; zoological: mean difference ± SD = 7.9 ± 10.0 kg, CI = 6.4–9.5 kg, *p* < .001, *d* = 0.79; Table 2). However, there was no sex difference in adult body mass within the research population (mean difference ± SD = 0.1 ± 9.9 kg, CI = −4.3–4.3 kg, *p* = .995; Table 2). The rate of growth did not differ between sexes in the sanctuary population; however, males in the zoological and research populations had a greater growth rate compared to their female counterparts (both *p* < .001; Table 3). The age of body mass maturation was not statistically different between sexes, for any population (Table 3).

## 4 DISCUSSION

The purpose of this study was to compare adult body mass, body mass growth rates, and the ages of body mass maturation between sanctuary, zoological, and research chimpanzees, and to compare these growth parameters between sexes, within each population. The main findings were (1) zoological and research chimpanzees were heavier than those living in sanctuaries; (2) male sanctuary chimpanzees had a slower rate of growth and attained maturation at an older age compared to male zoological and male research chimpanzees; (3) in females, sanctuary chimpanzees also had a slower rate of growth compared with their zoological and research counterparts, however the age at maturation was similar across the female populations; (4) no sex difference was observed for the growth rate in the sanctuary population; whereas, in zoological and research chimpanzees, males had a greater growth rate than females. These data contribute to the current understanding of growth and maturation in this species and suggest that growth patterns may vary between chimpanzees living in different captive environments.

### 4.1 Differences in adult body mass across captive populations

African sanctuary chimpanzees have previously been reported to have a lower body mass compared to research chimpanzees (Cole et al., 2020). Consistent with these findings, and in agreement with our hypothesis, the present study has also shown that adult body mass is lower in African sanctuary chimpanzees compared with research and zoological populations. Owing to limited information regarding the husbandry of the zoological and research populations, it is difficult to conclusively identify what factors may be influencing the findings of this study. However, several factors likely contribute to the variation in adult body mass across captive living environments, including physical activity and diet. It is possible that physical activity levels are lower in zoological and research chimpanzees compared with those in the African sanctuaries included in this study, due to enclosure size and environmental complexity. The enclosures at African sanctuaries are large, forested areas encouraging regular bouts of vertical climbing, arboreal travel, and foraging. In contrast, research enclosures are smaller than those of African sanctuaries and can lack three‐dimensional complexity, cognitive stimulation, and foraging opportunities, leading to general inactivity (Celli et al., 2003; Lewton, 2017; Paquette & Prescott, 1988). Whilst zoological institutions have developed robust enrichment programs (AZA Ape TAG, 2010) to increase physical activity of the chimpanzees (Zaragoza et al., 2011), and enclosures have evolved considerably in recent decades to become larger, open‐air spaces (Ross, 2014), it is logistically impossible to re‐create the size and complexity of the environment that many sanctuary animals experience. Future work should compare physical activity levels between sanctuary and zoological chimpanzees to confirm or refute whether differential opportunity for physical activity influences overall size or rates of growth in different captive populations.

Differences in diet and food availability across captive living environments may also contribute to the greater body mass in zoological and research populations, compared with sanctuary animals. For example, a staple portion of the zoological and research chimpanzee diet is commercial monkey biscuit (AZA Ape TAG, 2010), which likely provides greater caloric and lower fiber intake than the natural vegetation that sanctuary chimpanzees consume. Additionally, it is possible that portion size (i.e., the amount of food per chimpanzee) differs across the three populations, which could affect body mass. However, this information was not available across the three populations and so it is not possible to make this direct comparison. Body mass could also be influenced by the size of the chimpanzee's social group. Larger group sizes are associated with a complex social hierarchy, and lower‐ranking individuals may have reduced access to resources compared to more dominant individuals. Accordingly, body mass may be more variable amongst chimpanzees in African sanctuaries, which have much larger group sizes compared to those in zoological and research institutions, where within‐group competition is likely lower (Markham & Gesquiere, 2017). Accordingly, it is possible that zoological and research chimpanzees could have a more positive energy balance than sanctuary animals, which may explain the greater adult body mass we have described.

### 4.2 Differences in growth rate across captive populations

Environmental factors, such as diet (Jarrett et al., 2020) and the energetic costs related to physical activity and foraging (Zihlman et al., 2007), are believed to influence the rate of growth in primates. As discussed above, both diet and physical activity are likely to differ across captive living environments, which could result in a slower rate of growth in the sanctuary population. However, the influence of environmental factors on growth rate could be further exacerbated in sanctuary chimpanzees by their status as an orphan. Previous research in wild chimpanzees observed a lower muscle mass in orphans compared to nonorphaned individuals (Samuni et al., 2020). In their study, Samuni et al. (2020) proposed that the compromised growth in orphan chimpanzees could result from a need to allocate energy towards independent travel, foraging, and navigating a complex social hierarchy. In support of this, an exploratory analysis performed in our sanctuary population showed that despite no differences in either the age of body mass maturation or adult body mass, the rate of growth was slower in orphans (3.2 kg yr^−1^) compared to those who were sanctuary born (3.6 kg yr^−1^). Whilst this provides useful insight, the sample size of the sanctuary‐born cohort used in this exploratory analysis was relatively small and so further research is needed to confirm this finding.

### 4.3 Differences in the age at body mass maturation across captive living environments

Whilst we hypothesized that sanctuary chimpanzees would attain body mass maturation at an older age compared with their zoological and research counterparts, this was only supported in our male data. Aforementioned factors, such as physical activity, diet, and resource competition, are likely to be related to the comparatively longer growth period in sanctuary males. In contrast, we can only speculate as to why the age at maturation was similar in females across captive living environments. Chimpanzees often arrive at the sanctuaries malnourished and/or dehydrated (Wobber & Hare, 2011) and have experienced early‐life stress which, at a young age, may have long‐term implications on growth (Martins et al., 2011). These environmental stressors have been shown to affect growth more adversely in males than females (Semproli & Gualdi‐Russo, 2007), and could contribute to our findings. A similar sex‐dependent relationship has also been observed in humans and rodents, whereby poor nutrition was associated with a greater delay in puberty in males than in females (Kulin et al., 1982; Sánchez‐Garrido et al., 2013). However future investigation is required to assess whether a similar sex‐dependent relationship is present in chimpanzees.

### 4.4 Sexual dimorphism

In primates, body mass dimorphism (i.e., that males are heavier than females) can either arise through sex differences in the duration and/or rate of growth (Setchell et al., 2001). However, Leigh and Shea (1996) have proposed that, in chimpanzees, body mass dimorphism is caused by differences in the rate of growth and no sex differences in growth duration. Whilst our data support this hypothesis in zoological and research populations, no sex differences were observed for growth rate in the sanctuary population. Duration of growth, therefore, may have a comparably greater effect on sexual dimorphism in the sanctuary population. This is supported by the finding that males were estimated to attain body mass maturation approximately one and a half years after females; although this did not reach statistical significance according to conventional analysis. It is possible that this prolonged growth of sanctuary males reflects greater intermale resource competition compared to that in zoological or research institutions, which could result from their larger group size, as has been proposed in other primate species (Leigh & Shea, 1996).

### 4.5 Study limitations

The piecewise linear regression method adopted in this study was beneficial for identifying the estimated ages at maturation, but it does provide a simplistic view of growth rates by assuming they are constant. Alternative methods, such as pseudo velocity curves (Hamada & Udono, 2002), can visually demonstrate how growth rates fluctuate with age, but cannot be used for statistical comparison. Additionally, the body mass measurements of research chimpanzees used in this analysis were collected between 1980 and 2011. During this time, husbandry practices in research institutions have likely changed which may have affected the growth of the animals. Consequently, it is possible that the body mass observed for the research chimpanzees is not wholly reflective of current husbandry practices. Moreover, due to a paucity of information regarding the zoological and research populations, the authors were unable to provide information about how often the chimpanzees were weighed which may have influenced growth. To reduce the confounding effects of this unknown variable, we randomly selected only one measurement per individual. Furthermore, the authors have used the North American guidelines for chimpanzee care as a reference for husbandry practices in zoological institutions. However, we acknowledge that European and North American zoological practices may vary, but at present, European guidelines for the care of chimpanzees do not exist. Finally, reproduction and its associated costs (i.e., gestation and lactation) will influence the growth pattern of female chimpanzees. However, the authors were unable to determine its effects in this study as detailed information is not available across all of our populations.

## 5 CONCLUSION

This study contributes to our current understanding of chimpanzee growth and highlights that growth patterns may vary between chimpanzees living in different captive environments. Chimpanzees in African sanctuaries have a lower body mass than those in zoological and research facilities and a slower growth rate than their research counterparts. Additionally, male sanctuary chimpanzees also had a delayed body mass maturation compared to their zoological and research counterparts, whereas the age of maturation was similar across female populations. These results provide a valuable perspective regarding the influence of living environment on growth and suggest that caution should be observed when extrapolating growth parameters across different captive environments.

## CONFLICT OF INTEREST

The authors declare no conflict of interest.

## Data Availability

The data from the sanctuary population of chimpanzees which support the findings of this study are available upon reasonable request from the corresponding author. The data from the zoological and research populations that support the findings of this study are available from Species360 and the Primate Aging Database, respectively. Data for the zoological and research populations are available from the corresponding author, with the permission of Species360 and the Primate Aging Database, respectively.
